# Characterizing Behavioral and Brain Changes Associated with Practicing Reasoning Skills

**DOI:** 10.1371/journal.pone.0137627

**Published:** 2015-09-14

**Authors:** Allyson P. Mackey, Alison T. Miller Singley, Carter Wendelken, Silvia A. Bunge

**Affiliations:** 1 Department of Brain and Cognitive Sciences, Massachusetts Institute of Technology, Cambridge, MA, United States of America; 2 Department of Psychology, University of California, Berkeley, Berkeley, CA, United States of America; 3 Helen Wills Neuroscience Institute, University of California, Berkeley, Berkeley, CA, United States of America; University College London, UNITED KINGDOM

## Abstract

We have reported previously that intensive preparation for a standardized test that taxes reasoning leads to changes in structural and functional connectivity within the frontoparietal network. Here, we investigated whether reasoning instruction transfers to improvement on unpracticed tests of reasoning, and whether these improvements are associated with changes in neural recruitment during reasoning task performance. We found behavioral evidence for transfer to a transitive inference task, but no evidence for transfer to a rule generation task. Across both tasks, we observed reduced lateral prefrontal activation in the trained group relative to the control group, consistent with other studies of practice-related changes in brain activation. In the transitive inference task, we observed enhanced suppression of task-negative, or default-mode, regions, consistent with work suggesting that better cognitive skills are associated with more efficient switching between networks. In the rule generation task, we found a pattern consistent with a training-related shift in the balance between phonological and visuospatial processing. Broadly, we discuss general methodological considerations related to the analysis and interpretation of training-related changes in brain activation. In summary, we present preliminary evidence for changes in brain activation associated with practice of high-level cognitive skills.

## Introduction

Fluid reasoning, the ability to solve novel problems, was once thought to be a fixed trait, stable across the lifespan and immutable to environmental factors. However, mounting evidence suggests that it comprises a set of skills that can be strengthened through instruction and/or practice [1–9]. Previously, we have reported changes in the structural and functional connectivity of the frontoparietal network following 100 hours of preparation for a standardized exam that involves reasoning skills (the Law School Admission Test, LSAT) [10,11]. Here, we investigate whether LSAT preparation, i.e., practice with complex reasoning problems, leads to improvements on transfer tasks of reasoning, and changes in neural recruitment during performance of these tasks.

Reasoning involves relational processing, or the identification of individual properties of complex stimuli, as well as relational integration, or the joint consideration of previously separate mental relations [12]. For example, to solve a transitive inference question such as “If Bill is taller than Dan, and Dan is taller than Matt, is Bill taller than Matt?”, it is necessary to process the relationships between Bill and Dan and between Dan and Matt in order to infer the relationship between Bill and Matt. The Analytical Reasoning subtest of the LSAT taxes relational processing and integration. As a simplified example, consider the following premises for ordering objects A through D: 1) A is before B, 2) C is before D, 3) C is not directly next to D, 4) B is not last, 5) A is not first. These premises need to be integrated to determine the correct order: CABD.

Tasks requiring relational integration rely on the close cooperation of several prefrontal and parietal regions [13–15]–in particular, the area around the intraparietal sulcus (IPS), rostrolateral prefrontal cortex (RLPFC), and, depending on the task demands, ventrolateral PFC (VLPFC) and/or dorsolateral PFC (DLPFC). Reasoning practice, then, could alter patterns of activation at one or more of these frontoparietal nodes [16], in addition to changing the connectivity between the nodes, as we have observed previously [10,11]. Further, reasoning practice could lead to a qualitative change in the brain regions involved in reasoning. For example, because LSAT instruction techniques focus on drawing spatial diagrams to tackle text-based problems, participants could shift their reasoning strategies from a verbal to a spatial approach, leading to shifts in the cortical resources brought to bear on reasoning tasks. Finally, because reasoning involves many cognitive processes in addition to relational processing and integration, reasoning practice could lead to changes in the interactions between the frontoparietal network and other networks.

Because reasoning relies on abilities such as perceptual processing, attention, and working memory, reasoning practice may lead to improvements in these supportive skills. However, evidence for this type of cross-transfer is mixed [16–21]. Further, the reasoning instruction paradigm selected for this study intentionally minimized the working memory demands of complex reasoning problems by teaching students to break problems into tractable pieces and write down intermediate steps. Because of the nature of the instructional strategies employed during LSAT preparation, we predicted that we would observe selective gains in relational reasoning, but were also interested in assessing the reach of transfer to other cognitive skills.

In the present study, we tested whether reasoning instruction led to improved performance on two reasoning tasks: a transitive inference task and a rule generation task. Both tasks included a condition that involved relational processing alone, and a condition that involved both relational processing and integration. Further, we investigated whether reasoning instruction was associated with changes in brain activation during performance of these tasks. Finally, we examined whether reasoning instruction transferred to measures of matrix reasoning, working memory, and processing speed. To our knowledge, this is the first study to examine the effects of reasoning instruction on task-related brain activation.

## Methods

### Ethics Statement

Research was approved by the Committee for the Protection of Human Subjects at the University of California, Berkeley. Written informed consent was obtained from all participants.

### Participants

Participants in the LSAT group were recruited from the Blueprint Test Preparation course—an intensive, effective course that prepares students for the LSAT. This course consisted of 100 hours of classroom time: 35 for Logical Reasoning, 35 for Analytical Reasoning, and 30 for Reading Comprehension. Logical Reasoning instruction focused on the rules of formal logic. Analytical Reasoning instruction taught students to integrate multiple rules to determine the sequence or arrangement of a group of items. Reading Comprehension instruction covered tips for answering questions about short passages. A sample test is available at http://www.lsac.org/docs/default-source/jd-docs/sampleptjune.pdf.

To control for the effects of participating in research at two time points, including practice effects on tasks, increased familiarity with the scanner environment, and developmental changes between time points, we also recruited a group of pre-law students who did not prepare for the LSAT between scanning sessions. Control group participants were recruited through pre-law organizations on campus and online postings. The control group was matched to the LSAT group on age, sex, IQ, and days between testing sessions (S1–S3 Tables).

During an initial screening session, participants confirmed that they had learned English before the age of five and did not have a history of psychiatric or neurological disorders. Participants completed the Adult Self Report [22] (no participants met clinical criteria) and the Wechsler Adult Scale of Intelligence (WASI) Vocabulary and Matrix Reasoning subtests [23]. Control group participants were included in the study if their IQ scores were within one standard deviation of the mean for the LSAT group. After the initial screening session, participants visited the lab twice: once within two weeks of the start of their LSAT preparation course, and once within two weeks after completing the course, with a similar delay period for the control group.

We excluded participants for the following reasons: more than 3 standard deviation change in self-reported stress (Perceived Stress Scale [24]) or sleep (mean number of hours per night over previous two weeks; 1 participant from each group excluded from all analyses), head motion (mean displacement between volumes) of more than 3 standard deviations above the average across tasks (Transitive Inference: no subjects excluded; Letter Series: 1 from control group, 2 from LSAT group), or poor performance on the task (Transitive Inference: 2 from control group, 1 from LSAT group; Letter Series: 1 from control group, 1 from LSAT group). Poor task performance was defined as performance at or below chance on responded trials (50% for Transitive Inference, 25% for Letter Series) in either session and in either condition. One participant was included in the behavioral analysis of the Transitive Inference task but not the imaging analysis, because only one run was usable at time 2 (fingers slipped to the wrong buttons in the middle of the second run). Participants with excessive head motion were included in behavioral analyses. Transitive Inference data were available for 24 LSAT participants (23 with usable imaging data), and 22 control participants. Letter Series data were available for 17 LSAT participants (15 with usable imaging data), and 23 control participants (22 with usable imaging data). Demographic details for the participants included in behavioral and neuroimaging analyses are presented in S1–S3 Tables. Group sizes for each behavioral measure are described in the legend of Table 1.

**Table 1:** Behavioral Measures. Data are presented as *M*(*SD*). Reading Span is Absolute Span. Cross Out data are correct items per second. LSAT Group: Cattell *n* = 25, Digit Span *n* = 18, Spatial Span *n* = 25, Reading Span *n* = 25, Cross Out *n* = 17. Control Group: Cattell *n* = 24, Digit Span *n* = 23, Spatial Span *n* = 24, Reading Span *n* = 24, Cross Out *n* = 17.

		LSAT		Control	
		Time 1	Time 2	Time 1	Time 2
Cattell		28.6 (3.0)	29.7 (5.1)	27.8 (4.4)	29.2 (4.0)
Digit Span	Forward	11.2 (2.0)	11.8 (2.0)	11.5 (1.9)	11.3 (2.8)
	Backward	8.4 (2.4)	8.5 (2.3)	8.1 (2.5)	7.8 (2.0)
Spatial Span	Forward	9.8 (1.6)	9.8 (1.9)	9.3 (2.0)	9.3 (1.7)
	Backward	8.6 (1.7)	8.6 (1.8)	8.8 (1.6)	8.7 (1.2)
Reading Span*		39.5 (14.8)	44.7 (16.1)	37.9 (15.4)	43.2 (15.7)
Cross Out		.17 (.02)	.18 (.03)	.17 (.02)	.18 (.02)

*LSAT group t-test is significant (*t*(24) = 2.2, *p* = .04).

### MRI Data Collection

Scanning was performed on a Siemens 3T Trio at the Brain Imaging Center at the University of California at Berkeley. Participants underwent a series of scans in the same order for every session. The scanning session included a high-resolution structural scan, the Transitive Inference task, a resting-state scan [10], the Letter Series task, and a diffusion-weighted imaging scan [11]. Both functional tasks were acquired with the same gradient-echo echo-planar imaging (EPI) sequence (TR = 2000ms, TE = 25ms, 33 axial slices, 2.0×1.8×3.0 mm voxels, no interslice gap, flip angle = 90°, field of view = 230mm). The Transitive Inference task was collected in 2 runs of 180 volumes each, and the Letter Series task was collected in one run of 165 volumes.

### Transitive Inference

**Fig 1:**
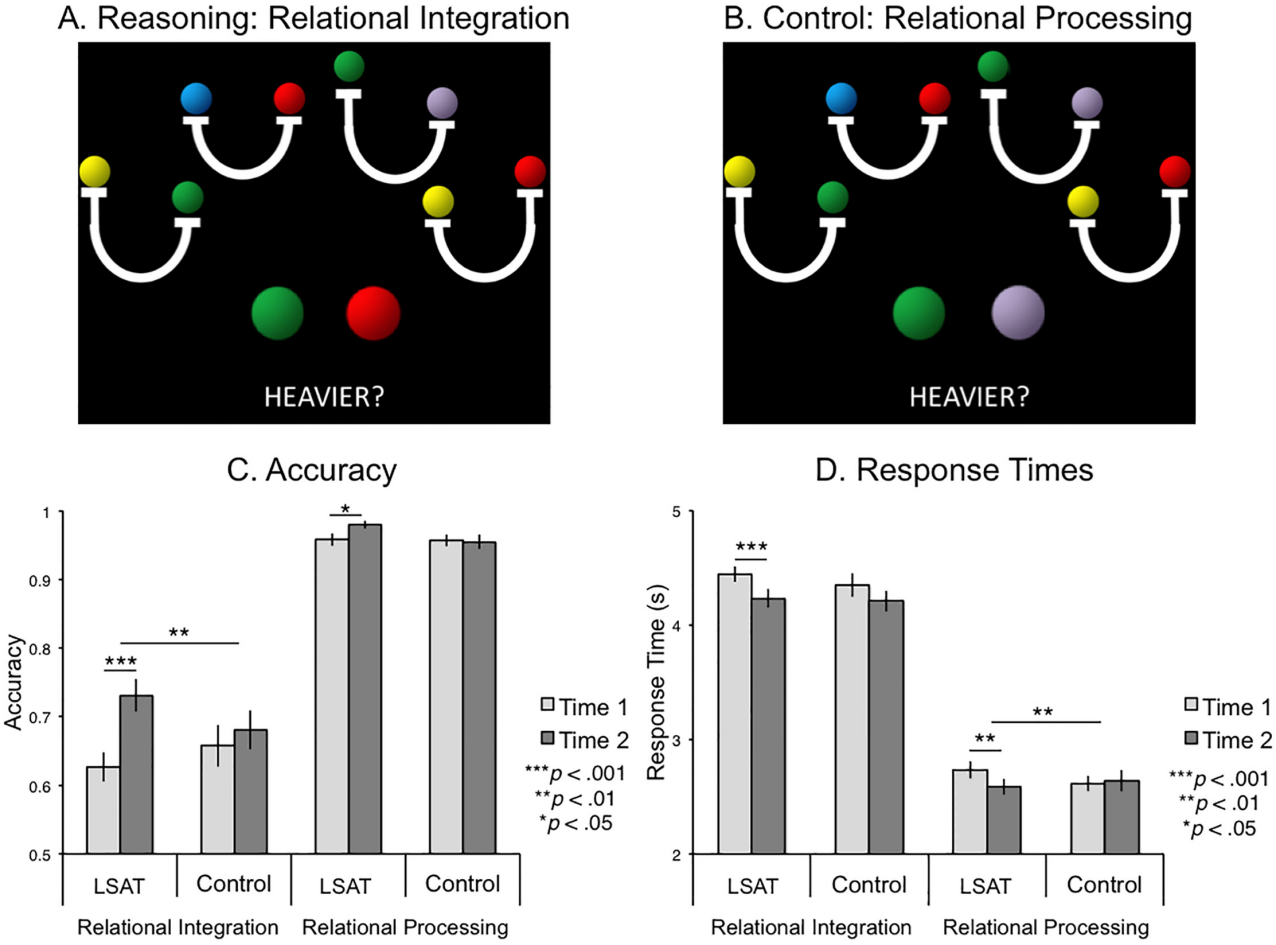
Transitive Inference task and behavioral results. (A) Sample Relational Integration Trial: The scale on the far left indicates that green is heavier than yellow, and the scale on the far right indicates that yellow is heavier than red. Therefore, the answer to the question is the left button (pressed with the index finger) because green is heavier than red. (B) Sample Relational Processing Trial: The scale that is second from the right indicates that purple is heavier than green, so the answer is the right button, pressed with the middle finger. (C) Accuracy. (D) Response times for correct trials.

In each trial of this task (modified from [25]), the participant was presented with four “balance scales” that indicated the relationship between two colored balls. The participant was asked to make a judgment regarding which of two target balls was heavier, and to press with his/her right hand index finger if the ball on the left was heavier, and his/her middle finger if the ball on the right was heavier. In the Relational Integration condition, it was necessary to integrate relationships provided by two of the four scales (Fig 1A). In the Relational Processing condition, the visual information was the same, but answering these questions required referencing only one scale (Fig 1B). This task was presented in an event-related design. Participants were given up to 6 seconds to answer. Jittered ITIs ranged from 0 to 8 seconds and accounted for 30% of total scan time.

### Letter Series Task

**Fig 2:**
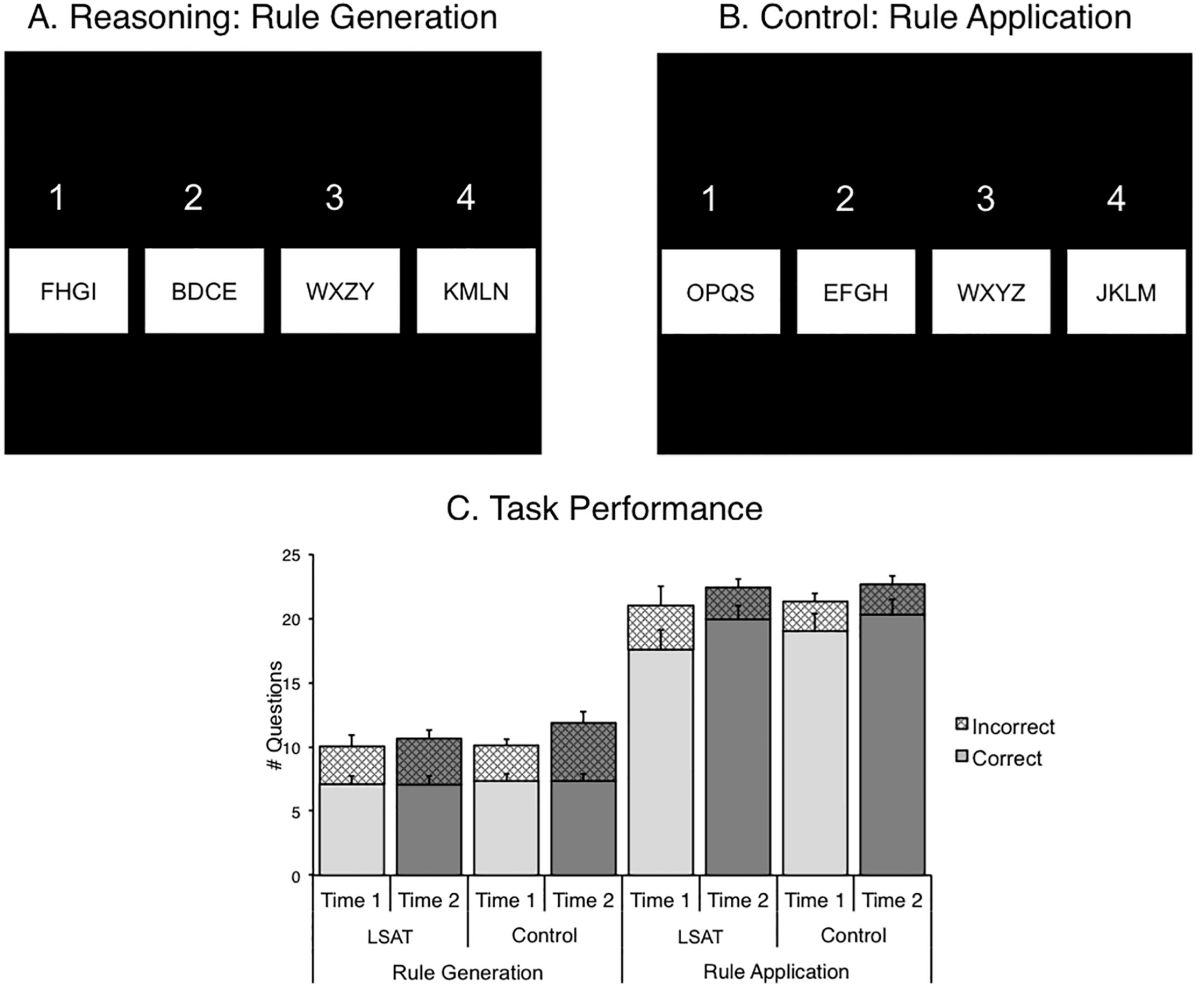
Letter Series task and behavioral results. (A) Sample Rule Generation Trial: Three of the four series are in alphabetical order, but with the middle two letters switched in order. Choice 3 does not follow this rule, so it is the correct answer (pressed with the ring finger). (B) Sample Rule Application Trial: Choice 1 is not in sequential alphabetical order (missing "R"), so it is the correct answer (pressed with the index finger). (C) Task Performance: Number of trials answered correctly (solid, bottom of each bar) and number of trials answered incorrectly (hatched, top of each bar). The LSAT group answered significantly more Rule Application questions correctly at time 2 compared to time 1 (*p* < .05). The control group answered significantly more Rule Generation questions incorrectly at time 2 compared to time 1 (*p* < .05). No other measures changed significantly.

The Letter Series task [26] required reasoning about sequences of letters. The Rule Generation condition required participants to discover a rule common to three of four letter strings, and to identify the string that did not follow this rule (Fig 2A). The rule changed for every trial. Rules were based on alphabetical order (e.g., skip two letters), orthographic features (e.g., straight or curved lines), or consonant/vowel categorizations. The rules became progressively more complex over the course of the session. In the Rule Application condition of the task, participants were asked to identify which of four letter strings was not in strict alphabetical order (i.e., missing a letter) (Fig 2B). At time 2, new stimuli were constructed following the same rules as the items at time 1, but using different letters so that participants would not remember the rules. The two conditions were completed in alternating 30s blocks of self-paced trials for a total of five minutes. Participants were instructed to respond only when they were confident that they were correct. Once a response was recorded, the next trial was presented without delay. Between blocks, a 3s cue was presented to instruct them of the condition of the next block.

### Behavioral Assessments

Behavioral testing occurred at both time points. We included three tests of working memory: Digit Span (Wechsler Adult Intelligence Scale [27]), Computerized Spatial Span (Lumos Labs), and Reading Span [28], a complex working memory measure that involved holding a series of letters in mind while judging whether sentences made sense (we report Absolute Reading Span). We also administered a test of spatial reasoning, the Cattell Culture Fair Intelligence Test III [29]. This test is a set of four timed tasks in which participants select pictures that complete an array, match an inferred rule, or have the same relationship to a prompt item. We chose this test because it contains two versions that could be counterbalanced across sessions by subject, and because it is sufficiently challenging that adult participants would not be expected to perform at ceiling. The two versions, A and B, were counterbalanced across time points, i.e., half of the participants took version A at time 1 and version B at time 2, and the other half took version B at time 1 and version A at time 2. Finally, we collected a measure of processing speed, Cross Out (Woodcock-Johnson III [30]), for a subset of participants.

### FMRI Data Analysis

FMRI data preprocessing was consistent across both tasks, and was carried out using FEAT (FMRI Expert Analysis Tool) Version 6.00, part of FSL (FMRIB's Software Library, www.fmrib.ox.ac.uk/fsl). The following preprocessing steps were applied: motion correction using MCFLIRT [31], slice-timing correction using Fourier-space time-series phase-shifting, non-brain removal using BET (Brain Extraction Tool [32]), spatial smoothing using a Gaussian kernel of FWHM 5mm, grand-mean intensity normalization of the entire 4D dataset by a single multiplicative factor, and high-pass temporal filtering (Gaussian-weighted least-squares straight line fitting, with sigma = 50.0s). Functional data were registered to anatomical space using FSL’s implementation of Boundary-Based Registration (BBR) [33]. Anatomical images were normalized to MNI standard space using linear registration, FLIRT [31,34].

For both tasks, the following covariates were included as nuisance regressors in the subject-level general linear models (GLMs): six motion parameters, average white matter signal, average cerebrospinal fluid signal, and average out-of-brain signal. Time-series statistical analysis was carried out using FILM with local autocorrelation correction [35].

For the event-related Transitive Inference task, the following behavioral regressors were included: correct Relational Processing trials, correct Relational Integration trials, incorrect Relational Integration trials, and omitted Relational Integration trials. The duration of each trial was convolved with a double-gamma hemodynamic response function (HRF). We also included temporal derivatives of these regressors. Incorrect and omitted Relational Processing trials were not modeled because they were so infrequent. The two runs of the task were combined in a fixed-effects analysis for each subject. For the blocked Letter Series task, we included regressors for Rule Generation and Rule Application blocks, convolved with a double-gamma HRF. We also included temporal derivatives of these regressors.

For both tasks, we calculated the difference between time 1 and time 2 with a fixed-effects analysis for each subject. These difference images were submitted to mixed-effects analyses (FLAME1+2) to test for: 1) average activation across groups and times; 2) changes between time 1 and time 2 for each group; 3) between-group differences in change between time 1 and time 2 (group × time ANOVA). Z (Gaussianised T/F) statistic images were thresholded using clusters determined by *Z* > 2.3 and a corrected cluster significance threshold of *p* = 0.05 [36]. For visualization, results were registered to a standard template in Freesurfer 5.3 (fsaverage) [37,38].

Contrasts of parameter estimates were extracted for each subject at both time points from the clusters identified from the whole-brain analyses. Parameter estimates were averaged within group and within time point. T-tests were conducted between groups at time 1. Parameter estimates defined from the whole-brain group × time ANOVAs were subjected to separate t-tests within both the LSAT group and control group to determine whether the results were driven by changes in one or both groups. In addition, parameter estimates defined from the group x time ANOVAs were submitted to group x time analyses of covariance (ANCOVAs) controlling for time 1 values, to investigate whether these interactions were driven by group differences at time 1.

## Results

### Reasoning Tasks: Behavioral Results

On the Transitive Inference task, the LSAT group improved significantly on Relational Integration trial accuracy between time 1 and time 2 (Fig 1C; *t*(23) = 5.29, *p* < .0001), and improved significantly more than the control group (group × time ANOVA *F*(1,44) = 7.7, *p* = .008). The LSAT group also improved significantly on Relational Processing trial accuracy (*t*(23) = 2.59, *p* = .02), but not significantly more than the control group (*F*(1,44) = 3.0, *p* = .09), likely due to ceiling effects. The effect of group on Relational Integration accuracy improvement was significant even after controlling for Relational Processing accuracy improvement (*t*(43) = 2.26, *p* = .03), so the improvement in relational integration cannot be fully explained by improved processing of individual relations. The LSAT group responded significantly faster for correct responses at time 2 on both Relational Integration (*t*(23) = 3.72, *p* = .001) and Relational Processing trials (*t*(23) = 3.36, *p* = .003); however, only the improvement in average response time (RT) on the Relational Processing trials was significantly greater than in the control group (*F*(1,44) = 4.53, *p* = .04) (Fig 1D). There was a trend towards a correlation between decreased RTs and increased accuracy in both conditions (RI: *r*(22) = -.34, *p* = .1; RP: *r*(22) = -.34, *p* = .1), indicating that there was no speed-accuracy tradeoff. Further, the improvement in Relational Integration accuracy was significantly greater in the LSAT group than in the control group, even after controlling for changes in RT (*t*(43) = 2.6, *p* = .01).

The Letter Series task was self-paced, so the number of questions attempted varied across individuals. Therefore, the most informative behavioral measures were the numbers of correctly and incorrectly answered questions for each condition. The LSAT group answered more Rule Application questions correctly at time 2 than at time 1 (Fig 2C; *t*(16) = 2.37, *p* = 0.03) and the control group did not (*p* = .26), but this group difference was not significant (*p* = .48). Neither group changed significantly from time 1 to time 2 on the number of *incorrectly* answered Rule Application questions (*p*s > .2) or on the number of *correctly* answered Rule Generation questions (reasoning condition) (*p*s > .2). The control group answered significantly more Rule Generation questions *incorrectly* at time 2 compared to time 1 (*t*(22) = 2.63, *p* = 0.02), but the LSAT group did not change significantly on this measure (*p* = .5). There was no significant difference between groups on change in the number of incorrect Rule Generation questions (*p* = .33).

### Reasoning Tasks: Neuroimaging Results

**Fig 3:**
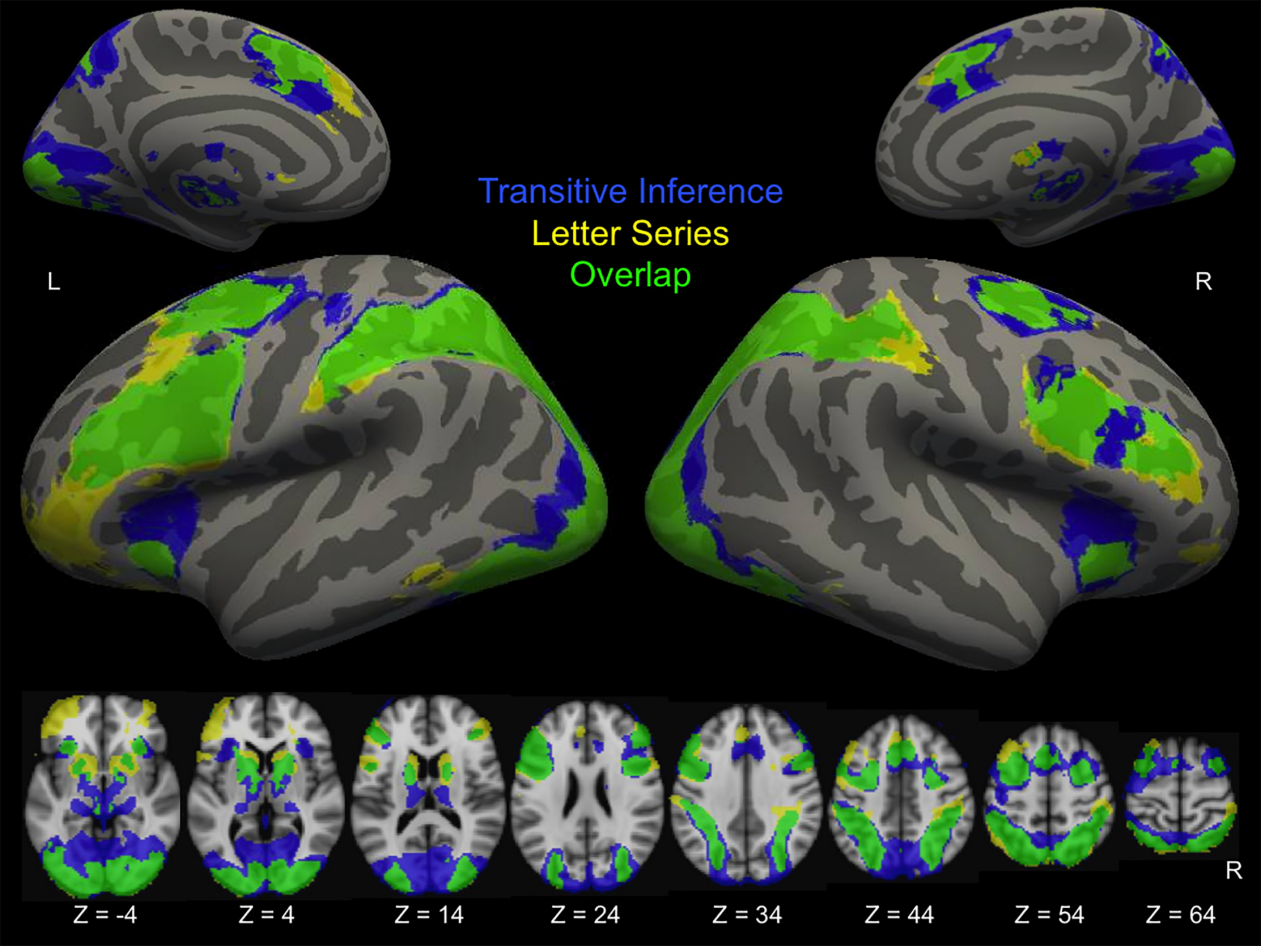
Task activation. Activation is averaged across groups and times for the reasoning condition of each task compared to implicit baseline. Voxels that were significant only in the Transitive Inference task are shown in blue. Voxels that were significant only in the Letter Series task are shown in yellow. Overlap is shown in green. Statistical maps are corrected for multiple comparisons at *Z* > 2.3, *p* < .05. Results were registered to the fsaverage template in Freesurfer, and displayed on inflated surfaces.

Across both groups and both time points, the conditions of both tasks that placed the strongest demands on reasoning (Relational Integration trials in the Transitive Inference task and Rule Generation trials in the Letter Series task) engaged partially overlapping areas of bilateral prefrontal, parietal, and occipital cortices, namely RLPFC, DLPFC, VLPFC, dorsal anterior cingulate, intraparietal sulcus, lateral occipital cortex, and lingual gyrus (Fig 3). Both tasks additionally engaged areas of the striatum and thalamus.

**Fig 4:**
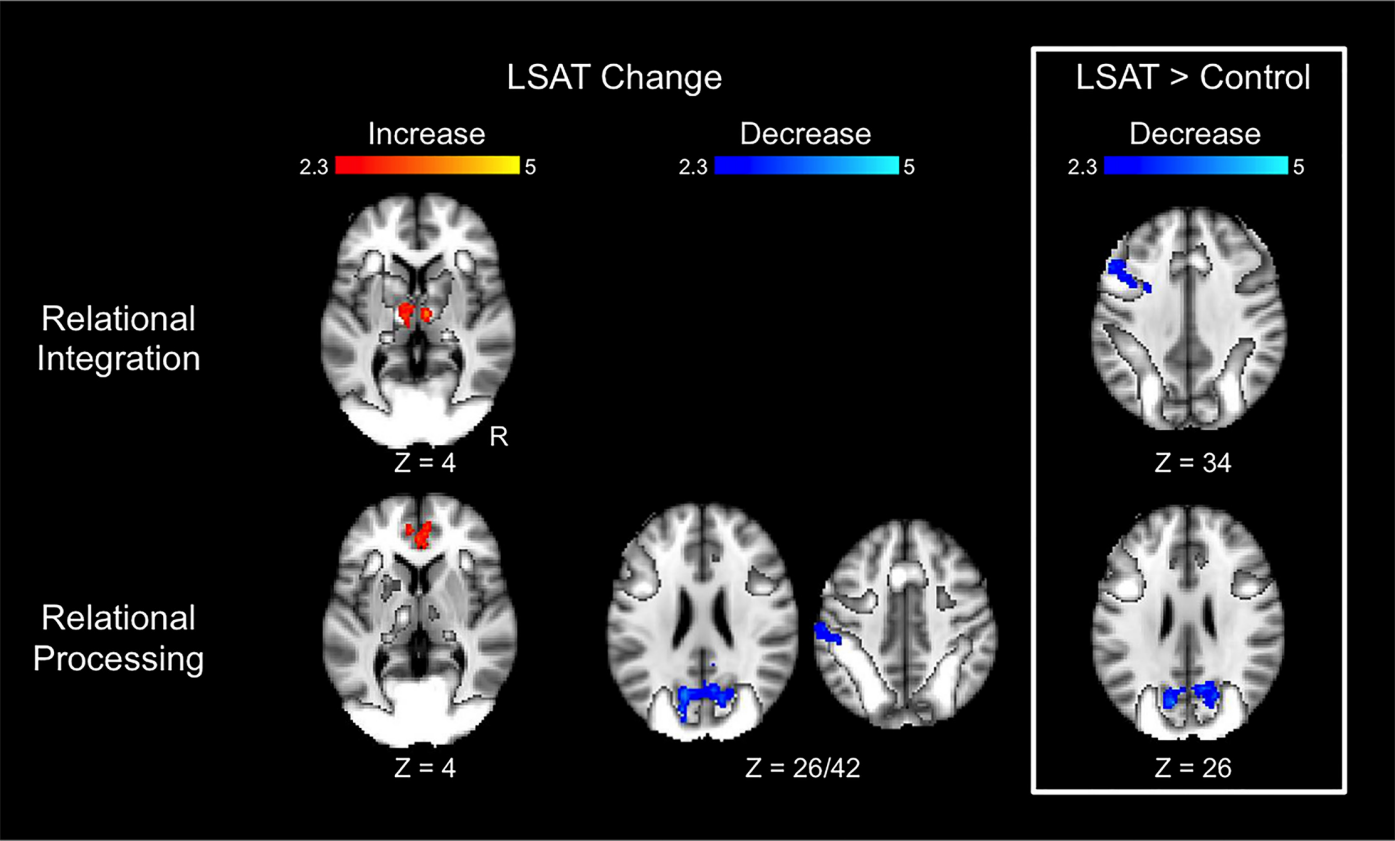
Changes in Transitive Inference task activation. Results are overlaid on the task activation observed across time points and across groups. Statistical maps show results of whole-brain analyses and are corrected for multiple comparisons at *Z* > 2.3, *p* < .05. Parameter estimates for these clusters are shown in Table 2.

Transitive Inference results are shown in Fig 4 and Tables 2–3. In the Relational Integration condition, the LSAT group exhibited increased activation from time 1 to time 2 in bilateral thalamus. The group × time ANOVA revealed a cluster in left middle frontal gyrus (DLPFC) that exhibited a significantly greater decrease in activation for the LSAT group than for the control group. This group difference was specific to the Relational Integration condition: parameter estimates for the Relational Processing condition extracted from this cluster did not show a change between time points (*t*(22) = .25, *p* = .8). In the Relational Processing condition, the LSAT group exhibited increased activation in anterior cingulate cortex, decreased activation in left supramarginal gyrus, and decreased activation in precuneus from near zero to more negative values. The precuneus decrease for the LSAT group was also significant in the whole-brain group × time ANOVA. Parameter estimates for the Relational Integration condition extracted from this cluster show a slight change between time points (R precuneus: *t*(22) = 2.4, *p* = .02; L precuneus: *t*(22) = 2.6, *p* = .02). Whole-brain analyses for the control group revealed no significant changes for either condition of the Transitive Inference task.

**Table 2:** Transitive Inference cluster locations.

Condition	Contrast	Direction	Region	Voxels	Center of Gravity (MNI)		
					X	Y	Z
Relational Integration	LSAT change	Increase	L and R thalamus	382	0	-14	5
	LSAT > Control	Decrease	L middle frontal gyrus	290	-46	17	31
Relational Processing	LSAT change	Increase	L and R anterior cingulate	297	1	44	0
	LSAT change	Decrease	L and R precuneus, L and R cuneus	908	2	-65	19
	LSAT change	Decrease	L supramarginal gyrus	253	-55	-25	43
	LSAT > Control	Decrease	R precuneus	269	12	-66	28
			L precuneus	246	-12	-70	23

**Table 3:** Transitive Inference cluster statistics. ° clusters significantly differed between groups at time 1 (*p* < .05). LSAT *t* and Control *t* statistics resulted from paired t-tests comparing Time 1 to Time 2 for each group. ANCOVA *F* statistics show the impact of group on activation change controlling for activation at time 1.

Condition	Contrast	Direction	Region	LSAT		Control		LSAT *(t)*	Control *(t)*	ANCOVA *(F)*
				Time 1	Time 2	Time 1	Time 2			
Relational Integration	LSAT change	Increase	L and R thalamus	1.25	18.70	12.80	20.10	†	2.1*	*N/A*
	LSAT > Control	Decrease	°L middle frontal gyrus	46.22	31.09	24.18	39.11	-4.02***	3.67**	-22.93***
Relational Processing	LSAT change	Increase	°L and R anterior cingulate	-58.97	-38.96	-46.54	-46.93	†	*n*.*s*.	*N/A*
	LSAT change	Decrease	L and R precuneus, L and R cuneus	0.62	-23.19	-11.19	-9.16	†	*n*.*s*.	*N/A*
	LSAT change	Decrease	L supramarginal gyrus	35.74	15.07	22.70	15.18	†	*n*.*s*.	*N/A*
	LSAT > Control	Decrease	°R precuneus	-8.30	-28.52	-26.14	-13.58	-6.70***	2.63*	-25.13***
			°L precuneus	-7.92	-31.64	-23.73	-11.44	-5.44***	2.34*	-21.05***

* *p* < .05,

** *p* < .01,

***p < .001.

*n*.*s*. = not significant,

^†^ = result determined by ROI-defining contrast,

N/A = not applicable due to bias by ROI-defining contrast.

Note that all follow-up tests of the LSAT > Control ROIs are biased by the ROI-defining contrast, but nevertheless provide critical insight into the observed interactions.

Table 3 shows results of follow-up analyses on parameter estimates extracted from clusters identified at the whole-brain level for the Transitive Inference task. First, for the follow-up tests of control group change within regions identified by change in the LSAT group, we observed the following: thalamus, which demonstrated increased activation for Relational Integration in the LSAT group, demonstrated a similar increase in the control group; in contrast, anterior cingulate and parietal regions that demonstrated changes in the LSAT group for Relational Processing did not demonstrate significant change in the control group. Second, for the follow-up tests on regions identified by the group x time ANOVA, we observed that the decreases for the LSAT group relative to controls were driven both by decreases within the LSAT group and also by smaller increases within the control group. Further, ANCOVAs controlling for time 1 activation revealed that these interactions were not driven by time 1 differences. Although these latter analyses are necessarily biased (i.e., the *p*-values are inflated), they serve to clarify the specificity of changes in the LSAT group and to exclude changes driven by the control group or by time 1 differences between groups.

**Fig 5:**
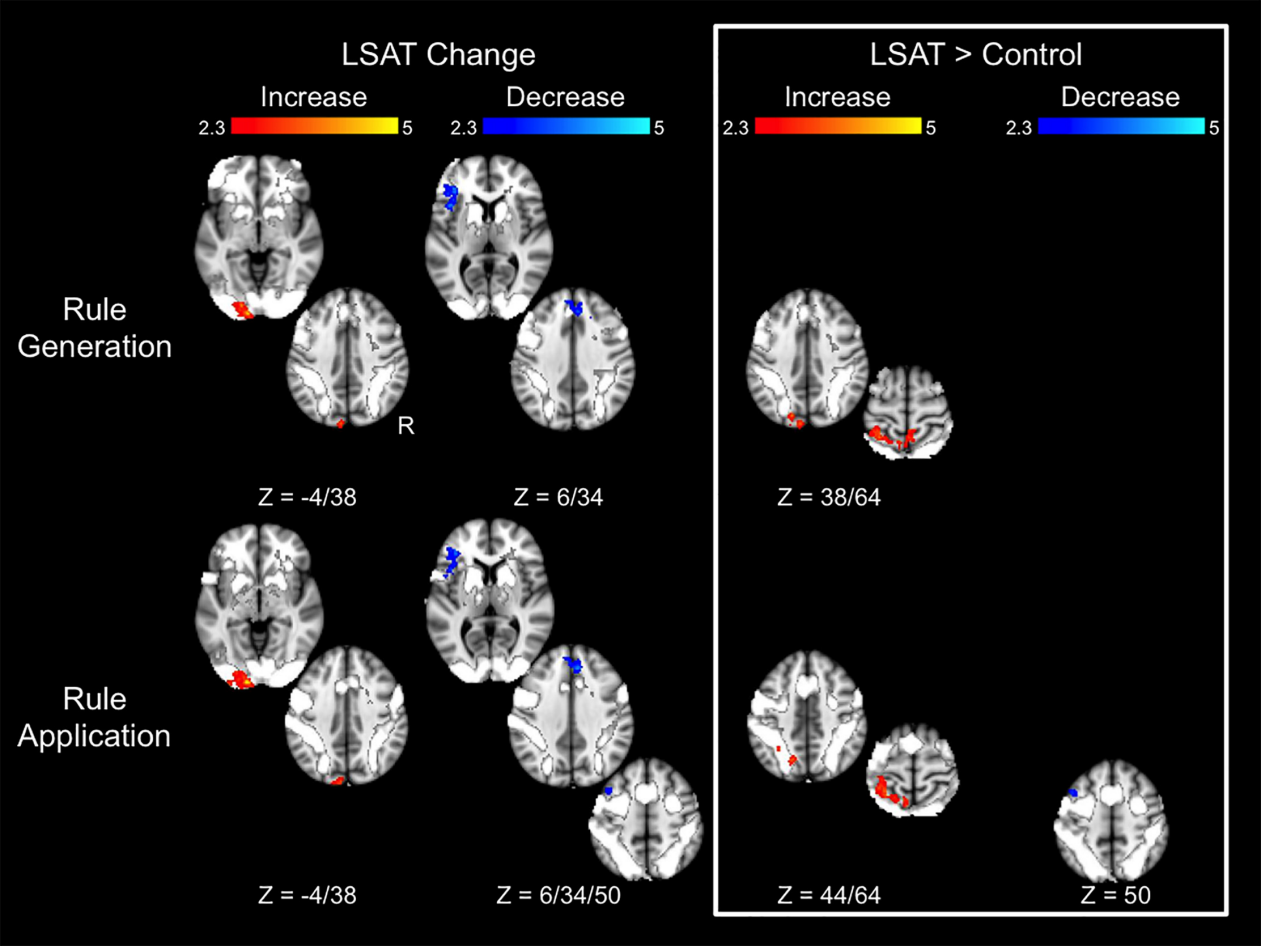
Changes in Letter Series task activation. Results are overlaid on the task activation observed across time points and across groups. Statistical maps show results of whole-brain analyses and are corrected for multiple comparisons at *Z* > 2.3, *p* < .05. Parameter estimates for these clusters are shown in Table 4.

Letter Series results are shown in Fig 5 and Tables 4–5. In the Rule Generation condition, the LSAT group showed increased activation in left occipital cortex. The superior aspect of this cluster was observed in the group × time ANOVA, as were regions in the left superior parietal lobule and right precuneus. The LSAT group also showed decreased activation in task-positive left inferior frontal gyrus (VLPFC) and dorsal medial prefrontal cortex. Changes in activation in the Rule Application condition appeared quite similar to those observed for Rule Generation: the LSAT group showed an increase in left occipital activation and decreases in left inferior frontal gyrus and dorsal medial prefrontal cortex activation. Increased superior parietal activation was observed in the group × time ANOVAs of both conditions. A region within left lateral prefrontal cortex (superior frontal gyrus/middle frontal gyrus) showed a decrease both in the LSAT t-test and in the group × time ANOVA for the Rule Application condition. Activation changes for the Rule Generation condition extracted from the clusters identified from the Rule Application condition were significant (L SPL: *t*(14) = 2.7, *p* = .02; L MFG/SFG: *t*(14) = 3.7, *p* = .002), as were changes for the Rule Application condition in clusters identified from the Rule Generation condition (L SPL: *t*(14) = 2.2, *p* = .05; R precuneus: *t*(14) = 3.1, *p* = .009; L occipital pole/L LOC: *t*(14) = 4.77, *p* = .0003). Whole-brain analyses for the control group revealed a decrease in superior parietal lobule activation for both conditions, as well as a decrease in left supramarginal gyrus and increase in left occipital pole activation for the Rule Application condition.

**Table 4:** Letter Series cluster locations.

Condition	Contrast	Direction	Region	Voxels	Center of Gravity (MNI)		
					X	Y	Z
Rule Generation	LSAT Change	Increase	L occipital pole, L lateral occipital cortex	721	-15	-98	8
	LSAT Change	Decrease	L frontal operculum, L inferior frontal gyrus	524	-41	26	1
	LSAT Change	Decrease	L and R paracingulate gyrus	323	7	36	29
	LSAT > Control	Increase	L superior parietal lobule	352	-24	-50	63
	LSAT > Control	Increase	R precuneus	257	6	-51	68
	LSAT > Control	Increase	L occipital pole, L lateral occipital cortex	236	-11	-86	36
	Control change	Decrease	L superior parietal lobule	259	-19	-57	61
Rule Application	LSAT Change	Increase	L occipital pole	694	-19	-99	0
	LSAT Change	Increase	L lateral occipital cortex	249	-3	-81	49
	LSAT Change	Decrease	L frontal operculum, L inferior frontal gyrus	550	-45	21	6
	LSAT Change	Decrease	L superior frontal gyrus, L middle frontal gyrus	341	-20	18	63
	LSAT Change	Decrease	L and R paracingulate gyrus	282	5	38	29
	LSAT > Control	Increase	L superior parietal lobule	572	-25	-50	60
	LSAT > Control	Decrease	L superior frontal gyrus, L middle frontal gyrus	283	-25	18	60
	Control change	Increase	L occipital pole	293	-21	-99	2
	Control change	Decrease	L superior parietal lobule, L precentral, L lateral occipital	509	-16	-51	61
	Control change	Decrease	L supramarginal gyrus	367	-55	-32	42

**Table 5:** Letter Series cluster statistics. ° clusters significantly differed between groups at time 1 (*p* < .05). LSAT *t* and Control *t* statistics resulted from paired t-tests comparing Time 1 to Time 2 for each group. ANCOVA *F* statistics show the impact of group on activation change, controlling for activation at time 1.

Condition	Contrast	Direction	Region	LSAT		Control		LSAT *t*	Control *t*	ANCOVA *F*
				Time 1	Time 2	Time 1	Time 2			
Rule Generation	LSAT Change	Increase	°L occipital pole, L lateral occipital cortex	-3.42	29.28	19.37	27.01	†	*n*.*s*.	*N/A*
	LSAT Change	Decrease	°L frontal operculum, L inferior frontal gyrus	16.32	-16.82	2.73	1.19	†	*n*.*s*.	*N/A*
	LSAT Change	Decrease	L and R paracingulate gyrus	13.73	-11.93	6.26	1.91	†	*n*.*s*.	*N/A*
	LSAT > Control	Increase	°L superior parietal lobule	-16.25	6.59	2.10	-19.49	2.7*	-5.5***	16.33***
			R precuneus	-11.18	26.92	-1.11	-20.14	3.3**	-2.5*	18.66***
			L occipital pole, L lateral occipital cortex	-26.31	2.24	-7.70	-28.40	4.54***	-3.07**	22.9***
	Control change	Decrease	L superior parietal lobule	7.72	10.29	14.96	-11.25	*n*.*s*.	†	*N/A*
Rule Application	LSAT Change	Increase	L occipital pole	11.73	43.08	33.48	51.20	†	2.93**	*N/A*
	LSAT Change	Increase	°L lateral occipital cortex	-1.54	38.45	15.31	6.01	†	*n*.*s*.	*N/A*
	LSAT Change	Decrease	°L frontal operculum, L inferior frontal gyrus	25.51	-6.75	12.08	11.52	†	*n*.*s*.	*N/A*
	LSAT Change	Decrease	°L superior frontal gyrus, L middle frontal gyrus	20.43	-14.57	-3.07	0.17	†	*n*.*s*.	*N/A*
	LSAT Change	Decrease	°L and R paracingulate gyrus	8.12	-18.43	-6.22	-9.87	†	*n*.*s*.	*N/A*
	LSAT > Control	Increase	°L superior parietal lobule	-0.79	19.27	16.10	-6.57	2.52*	-5.84***	21.78***
	LSAT > Control	Decrease	°L superior frontal gyrus, L middle frontal gyrus	11.60	-20.66	-11.52	-0.96	-5.18***	*n*.*s*.	-7.59**
	Control change	Increase	L occipital pole	17.11	40.68	33.51	59.92	4.19***	†	*N/A*
	Control change	Decrease	L superior parietal lobule, L precentral, L lateral occipital	2.78	9.01	12.78	-14.36	*n*.*s*.	†	*N/A*
	Control change	Decrease	°L supramarginal gyrus	21.23	17.99	37.01	10.82	*n*.*s*.	†	*N/A*

* *p* < .05,

** *p* < .01,

***p < .001.

*n*.*s*. = not significant,

^†^ = result determined by ROI-defining contrast,

N/A = not applicable due to bias by ROI-defining contrast.

Note that all follow-up tests of the LSAT > Control ROIs are biased by the ROI-defining contrast, but nevertheless provide critical insight into the observed interactions.

Table 4 shows results of follow-up analyses on parameter estimates extracted from clusters identified at the whole-brain level for the Letter Series task. First, among the regions that demonstrated change within the LSAT group, only left occipital pole demonstrated corresponding changes in the control group. Second, in follow-up analyses of ROIs identified in the group x time ANOVA (all biased by ROI selection but nevertheless informative), the following ROIs met the criteria that they demonstrated significant increase within the LSAT group, were not primarily driven by changes in the control group (i.e., LSAT group changes were stronger than control group changes, if any), and the group x time interactions were significant after controlling for time 1 values: right precuneus and left occipital cortex (Rule Generation) and left middle/superior frontal gyrus (Rule Application). Left superior parietal lobule did not meet these criteria in either condition.

### Results of Behavioral Assessments

Training did not transfer to measures of matrix reasoning (Cattell Culture Fair III), working memory (Reading Span, Digit Span, Spatial Span), or processing speed (Cross Out) (Table 1). The only test to show evidence of a practice effect was Reading Span. Both groups improved from Time 1 to Time 2 (LSAT: *t*(24) = 2.2, *p* = .04; Control: *t*(23) = 1.6, *p* = .12), and the group × time ANOVA was not significant (*p* = .97). For the Cattell test, we found a significant order effect, i.e., the change between time points differed by version (*t*(48) = 2.71, *p* = .009). Participants who received version A at time 1 improved when they took version B at time 2 (mean gain 3.1 points, *SD* = 4.9), but participants who received version B at time 1 scored about the same when they took version A at time 2 (*M* = -.46, *SD* = 4.3).

## Discussion

Reasoning instruction led to improvement on an unpracticed test of transitive inference. After three months of intensive practice with reasoning problems, participants demonstrated faster processing of individual relations and more accurate relational integration. Preparation for the LSAT consisted of reading multiple rules, and grouping or sequencing items according to the rules. In contrast, the transitive inference task involved making quick judgments about pictures of colored balls. Therefore, the task improvements we observed demonstrated a considerable degree of transfer. This finding is noteworthy, as transfer to unpracticed tests of reasoning has been notoriously difficult to observe, not only in cognitive neuroscience studies [17,20], but also in the classroom [39].

Reasoning instruction did not transfer to rule generation as measured by the Letter Series task. There are many possible reasons for this null result, including insufficient statistical power because this task was collected for fewer participants than the other task, and/or individual differences in the propensity to persevere on challenging trials. Subjects knew that they could advance to the next trial as soon as they had responded, and differed in their compliance with the instruction to proceed only once they were certain of the correct answer. These individual differences, along with the small number of attempted trials, may have made this task insensitive to subtle behavioral gains. Alternatively, it may be that the Letter Series task is too far of a transfer task with respect to LSAT preparation. We conceptualize this task as involving relational integration as well as the ability to generate and evaluate possible rules. This latter ability, which involves inductive rather than deductive reasoning, was not practiced as part of the LSAT course. Therefore, although the LSAT course may have improved relational integration, as suggested by the improvement on the Transitive Inference task, this boost may have not been large enough to also improve Letter Series performance, especially if the cognitive bottleneck is in the rule generation process.

Our approach to characterizing training-related changes in reasoning task activation was to identify regions that showed a group by time interaction, as well as regions that showed a change within the LSAT group. Group by time interaction analyses tend to identify regions that show a change in the opposite direction in the control group. Indeed, we see this pattern in the data presented here. It is possible that these regions randomly showed initial differences at time 1, and regression to the mean in each group. For this reason, it is important to determine whether changes are significant after controlling for parameter estimates at time 1. Follow-up analyses on the parameter estimates extracted from whole-brain results are biased, but are meant to be exclusionary rather than confirmatory: clusters that did not meet the criteria outlined above are unlikely to reflect true changes associated with reasoning instruction.

For the Transitive Inference task, reasoning instruction was associated with decreased DLPFC activation relative to the change in the control group during the reasoning condition of the task. Training-related decreases in DLPFC activation have been interpreted as evidence of greater neural efficiency, or less cognitive effort [40–43]. This interpretation has been criticized as simply a reinterpretation of the data rather than a mechanism [44], but fMRI methodology does not permit the exploration of cellular mechanisms. Reasoning instruction was also associated with decreased precuneus activation during the control condition of the task. In contrast to the group difference in changes in task-positive DLPFC activation, a decrease in task-negative precuneus activation in the trained group can be interpreted as a greater suppression of a node of the default mode network (DMN). DMN suppression during task performance has been associated with attention to the task [45], and better cognitive functioning more broadly [46]. Intriguingly, DMN activation has been shown to increase—or, rather, become less suppressed—as tasks become highly practiced and less effortful [47]. Observing the reverse pattern, as we did here, suggests that the reasoning task has not become rote (which stands to reason, because it was not explicitly practiced during LSAT preparation), but rather that instruction was associated with an improved ability to harness cognitive resources.

Reasoning instruction was associated with increased recruitment of occipital cortex for both conditions of the Letter Series task, possibly reflecting increased top-down control of visual attention [48,49]. Decreases in medial and lateral prefrontal regions were also observed, which, as described above, could reflect a reduction in the effort involved for attentional control to achieve the same level of behavioral performance. Indeed, given prior analyses of resting-state functional connectivity for this dataset, in which we found increases in temporal coupling among distant brain regions as a result of reasoning instruction [10], it is plausible that the increased occipital activation stems from increased functional connectivity with control-related regions. Alternatively, it is possible that reasoning instruction drove a shift in the balance between visuospatial and phonological processing strategies. In other words, participants may have used visual imagery rather than rehearsing the alphabet to detect patterns within letters sequences. This *post hoc* interpretation is based on the combination of observed increases in occipital regions and concomitant decrease in left inferior frontal gyrus (VLPFC) activation observed for the LSAT group. Although we did not observe significant behavioral benefits associated with these neural changes, it is possible that the behavioral data were not as sensitive as the neural data, or that a strategy shift did not translate to performance gains.

Across these two tasks, we observed preliminary evidence of three types of brain changes: 1) greater neural efficiency (decreased activation in task-positive regions in both tasks), 2) greater suppression of task-irrelevant networks (decreased activation in task-negative regions in the Transitive Inference task), and 3) a change in the cortical regions involved, perhaps due to a change in strategy in the Letter Series task. Future research with larger sample sizes and a larger range of tasks will be necessary to confirm these patterns and investigate the relationships between brain changes and behavioral improvements.

We found that reasoning instruction did not transfer to measures of either simple or complex working memory. Because the working memory demands of complex reasoning problems were intentionally minimized by the course instructors, it is not surprising that we did not observe working memory gains here (but note that we have previously found that practicing visuospatial reasoning games transfers to improved spatial working memory in children [1]). We also did not find transfer to our measures of processing speed or matrix reasoning. However, the matrix reasoning results were inconclusive, given that the two versions of the Cattell Culture Fair task differed in difficulty. More generally, it is difficult to draw strong conclusions about transfer with only one test per cognitive ability [20,50]. Indeed, it is possible that transfer was broader than we could observe with our limited test battery. In this way, functional and structural brain imaging could provide clues as to which kinds of cognitive changes are neurobiologically plausible, informing the selection of cognitive assessments for follow-up studies of cognitive transfer.

There are two important caveats regarding the results we have presented here. First, the intensity and unique characteristics of the reasoning training paradigm limited the choice of control groups, and therefore, for this first study, we opted for a passive control group with well-matched demographics. Including a passive control group was critical to rule out explanations such as practice effects on the task, increased familiarity with the research environment (especially the MRI scanner), and developmental changes. To confirm that the observed effects were due specifically to reasoning instruction and not generally to participating in an intensive course, it will be necessary to conduct additional research with an active control group. The use of an active control group would also alleviate the concern that the controls may have participated in other, undocumented, activities that drove changes in brain and behavior. To confirm that observed changes were not due to pre-existing differences between the groups, future work will need to involve random assignment to either the trained or the control group. The second caveat is that we present several behavioral and neuroimaging measures, which presents a multiple comparisons problem. As such, the results should be treated as a complete, but preliminary, account of the data collected in this study. The purpose of this work is to inspire more specific predictions about the scope of neural and behavioral changes associated with real-world learning.

In conclusion, we showed that practice with reasoning problems led to improved performance on an unpracticed task of relational integration, and shifts in neural recruitment during reasoning tasks. We took the worthwhile and underutilized approach of including two functional tasks to examine the reach of learning. This study provides more evidence that the neural circuitry that supports reasoning is malleable in adulthood.

## Supporting Information

S1 Table Demographics for participants with behavioral data. Data are presented as *M*(*SD*).
(PDF)

S2 Table Demographics for participants with Transitive Inference neuroimaging data. Data are presented as *M*(*SD*).
(PDF)

S3 Table Demographics for participants with Letter Series neuroimaging data. Data are presented as *M*(*SD*).
(PDF)
