# Supplementary material for: Characterizing Behavioral and Brain Changes Associated with Practicing Reasoning Skills
Source: PLoS One. 2015 Sep 14;10(9):e0137627. doi: 10.1371/journal.pone.0137627 (PMC4569435; doi:10.1371/journal.pone.0137627)
Supplement: S2 Table — Data are presented as M(SD). (PDF) [file pone.0137627.s002.pdf]

	LSAT (n = 23)	Control (n = 22)
Age	22.21 (1.94)	21.49 (2.00)
Sex	11 M/ 12 F	9 M / 13 F
Time Difference	90.09 (16.77)	93.95 (25.68)
WASI Matrix Raw	29.00 (2.59)	29.73 (1.70)
WASI Vocabulary Raw	65.78 (5.79)	67.05 (3.64)

S2 Table. Demographics for participants with Transitive Inference neuroimaging data. Data are presented as *M*(*SD*).
