# Supplementary material for: Characterizing Behavioral and Brain Changes Associated with Practicing Reasoning Skills
Source: PLoS One. 2015 Sep 14;10(9):e0137627. doi: 10.1371/journal.pone.0137627 (PMC4569435; doi:10.1371/journal.pone.0137627)
Supplement: S3 Table — Data are presented as M(SD). (PDF) [file pone.0137627.s003.pdf]

| | **LSAT (*n* = 15)** | **Control (*n* = 22)** |
|---|---|---|
| Age | 21.64 (1.49) | 21.43 (2.06) |
| Sex | 5 M/ 10 F | 12 M/ 10 F |
| Time Difference | 97.93 (12.65) | 91.41 (23.47) |
| WASI Matrix Raw | 29.00 (2.98) | 29.68 (1.76) |
| WASI Vocabulary Raw | 67.2 (4.96) | 66.91 (3.78) |

**S3 Table. Demographics for participants with Letter Series neuroimaging data.** Data are presented as *M*(*SD*).
